# Characteristics of patients with cancer in European long-term care facilities

**DOI:** 10.1007/s40520-021-01972-2

**Published:** 2021-09-29

**Authors:** Emanuele Rocco Villani, Domenico Fusco, Laura Franza, Graziano Onder, Roberto Bernabei, Giuseppe Ferdinando Colloca

**Affiliations:** 1 Department of Geriatrics, Fondazione Policlinico Universitario A. Gemelli IRCCS, Università Cattolica Sacro Cuore, Largo Francesco Vito n°8, 00168 Rome, Italy; 2 Department of Emergency Medicine, Fondazione Policlinico Universitario A. Gemelli IRCCS, Rome, Italy; 3 Dipartimento di Scienze Radiologiche, Radioterapiche ed Ematologiche, Fondazione Policlinico Universitario A. Gemelli IRCCS, Rome, Italy

**Keywords:** Cancer, Nursing home, Polypharmacy, Pain, Supportive care

## Abstract

**Purpose:**

Up to 26% of residents in nursing homes (NHs) are affected by cancer. Their care represents a challenge, because NHs are not usually considered a setting focused on oncologic management and care. The aim of this paper is to describe socio-demographic and clinical features of patients with cancer residing in European NHs.

**Methods:**

Cross-sectional study based on data from the Services and Health for Elderly in Long TERm care (SHELTER) study. Participants were assessed through the interRAI-LTCF, which includes cancer assessment.

**Results:**

Among 4140 participants (mean age 83.4 years; female 73%), 442 (10.7%) had cancer. Patients with cancer had a higher prevalence of do-not-resuscitate directives compared to those without cancer (21.1% vs 16.5%, *p* = 0.019). Variables directly associated with cancer were male sex (adj OR 1.67, 95% CI 1.36–2.05), pain (adj OR 1.43, 95% CI 1.16–1.77), fatigue (adj OR 1.25, 95% CI 1.01–1.55), polypharmacy (adj OR 1.59, 95% CI 1.21–2.08) and falls (adj OR. 1.30, 95% CI 1.01–1.67). Dementia was inversely associated with cancer (adj OR 0.74, 95% CI 0.58–0.94). Symptomatic drugs such as opioids (23.5% vs 12.2, *p* < .001), NSAIDS (7.2% vs 3.9%, *p* = 0.001), antidepressants (39.1% vs 33.8%, *p* = 0.026) and benzodiazepines (40.3% vs 34.3, *p* = 0.012) were all prescribed more in participants with cancer compared to those without cancer.

**Conclusions:**

Cancer patients are prevalent in European NHs and they show peculiar characteristics. Studies are needed to evaluate the impact of a supportive care approach on the management of NHs residents with cancer throughout all its phases, until the end-of-life care

## Introduction

In literature, prevalence of cancer patients in European nursing homes (NHs) ranges from 1% in Belgium [1] to 14–26% in Norway [2]. NHs are not considered a usual setting for cancer nursing care, because this care is traditionally provided by hospitals or clinics. Yet, older patients with cancer can be admitted to NHs for several reasons, not always strictly related to cancer, such as comorbid conditions, chronic treatment or poor social support [3, 4]. Likewise, cancer can also be a comorbidity in a complex patient who is admitted to a NH for other reasons. It is also worth considering that in Europe between 1.5 and 8% of older adults live in NHs [5]. The main reasons for admittance into NHs are related to functional disabilities in the activities of daily living (ADL), dementia, stroke, cardiovascular disease and poor social support, and this population does indeed present a high prevalence of multi-morbidity, geriatric syndromes and frailty, whose management is complex [4]. Management and care of this population can be even harder when considering cancer, not only due to the disease itself, but also because of medical procedures and treatment-related toxicity. Such conditions can lead to supportive care needs, polypharmacy, decline in functional and cognitive status, onset of geriatric syndromes and depression. In general, older people with active cancer report poorer health related quality of Life (HRQoL) than older people with no history of cancer, due to the high comorbidity burden, physical and mental symptoms and treatment-related issues [6]. Interestingly, older cancer survivors report HRQoL comparable to the general population [7].

Considering the current and future demographics and health care trends in Europe, NHs will continue to provide care to cancer patients at different stages of the disease. As long-term care systems develop, focus should be put on providing high-quality cancer care, especially to those who are old and frail [8].

The present study aims to explore the clinical and socio-demographic characteristics of a sample of European NHs residents with cancer.

## Methods

### Study population

This is a multicentre cross-sectional study based on data from the Services and Health for Elderly in Long Term care (SHELTER) study, conducted between 2009 and 2011 [9]. SHELTER includes information on 4156 NH residents from 50 European facilities (ten in Czech Republic, nine in England, four in Finland, four in France, nine in Germany, ten in Italy and four in the Netherlands) and from seven facilities in Israel.

All participants were evaluated by trained assessors through the interRAI-LongTerm Care assessment tool (InterRAI-LTCF). The InterRAI-LTCF includes over350 elements, including socio-demographic characteristics, clinical items about physical and cognitive status, and clinical diagnoses. The tool also includes data about an extensive array of signs, symptoms, syndromes, and treatments provided, and it is used to evaluate the characteristics and management of patients admitted and managed in the majority of the NHs worldwide. Information on the validity and reliability of InterRAI-LTCF data has been published elsewhere [9].

Ethical approval for the study was obtained in all countries according to local regulations. Residents were invited to take part in the study and were free to decline participation. Consent was obtained with assurance of data confidentiality.

Thoroughness in drafting the current short report was assessed via the Strengthening The Reporting of OBservational Studies in Epidemiology (STROBE) checklist [10].

### Cancer

Diagnosis of cancer according to ICD-9 codes specific for malignancies was collected for all the participants, as part of the InterRAI-LTCF. InterRAI-LTCF reports cancer as: not present; primary diagnosis and/or diagnoses for current stay; diagnosis present and receiving treatment; diagnosis present and monitored without receiving treatment. Patients who had a past (> 5 years) history of cancer are reported as not present [11]. For the purpose of the present study, cancer has been codified into two groups: patients without cancer; patients with cancer (including primary diagnosis and/or diagnoses for current stay, diagnosis present and receiving treatment, diagnosis present without receiving treatment). 16 participants were excluded because data on cancer was missing.

### Cognitive and functional status

Functional status was assessed through the even-point ADL Hierarchy scale. The ADL Hierarchy scale ranges from 0 (no impairment) to 6 (total dependence) [12].*Cognitive function* was assessed through the Cognitive Performance Scale (CPS). CPS combines information on memory impairment, level of consciousness and executive function, with scores ranging from 0 (intact) to 6 (very severe impairment). CPS has been shown to correlate with the Mini Mental State Examination (MMSE) in several validation studies [12]. Depressive symptoms were assessed through the Depression Rating Scale included. A score ≥ 3 is indicative of depression. This scale is comparable to the Geriatric Depression Scale, when tested in an older patient with a psychiatric diagnosis [13].

### Covariates

Data on participants’ sex and age at baseline were retrieved from the InterRAI-LTCF questionnaire. Body mass index (BMI), indicator of nutritional status, was derived from height and weight for each participant. Self-rated health items in the interRAI-LTCF could be classified in five different categories: excellent, good, fair, poor and no response. Information on all the drugs the participants had been taking was collected, including “as needed” drugs, according to the Anatomical Therapeutic and Chemical codes [14]. The definitions of polypharmacy as the contemporary use of more than five drugs was chosen to make the study comparable to others present in literature [9]. Delirium was defined as acute change in mental status. Falls were defined as a sudden loss of balance, resulting in the contact of any part of the body above the feet with the floor and were considered when occurring in the 90 days before the assessment. Insomnia was defined as difficulty in falling asleep or staying asleep, waking up too early, restlessness, or non-restful sleep. Pain was defined as presence of pain signs (including grimacing, grinding teeth, defence reactions when touched or other non-verbal signs suggesting pain). Dizziness, dyspnoea, and fatigue were deemed present if occurring in at least once during the three days before the assessment.

### Statistical analysis

The baseline study sample characteristics were compared according to cancer diagnosis, and reported as mean ± standard deviation, or counts and proportions (%), as appropriate. Student’s *T* test and Pearson’s Chi-square test were used to compare the distribution of continuous variables and categorical variables, respectively. A two-tails *p* value < 0.05 was considered significant. To better define the association between cancer and other variables, a multivariable logistic regression analysis was performed. We evaluated the combined OR (Mantel–Haenszel test) of the variables with a *p* > 0.10 at the univariate analysis. Variables whose combined OR was 10% different from the crude OR, as well as demographics, ADLs and CPS scale, were considered as covariates. A sensitivity analysis was conducted to exclude those participants not receiving active cancer treatment. All analyses were performed using SPSS version 20.0 (IBM SPSS Statistics, Armonk, New York) for Windows version 20.0.

## Results

### Sample characteristics

The final sample consisted of 4140 participants with a mean age of 83.4 years and 73% were female. 442 (10.7%) had a diagnosis of cancer. Socio-demographic and clinical characteristics of the participants are presented in Table 1. Mean age of participants with and without cancer was comparable. 162 on 1115 (14.5%) male patients had cancer, while 280 on 3025 (9.3%) female patients had cancer. Also, prevalence of cancer participants significantly differed between countries, ranging from 7.2% in Finland to 19.5% in France.

**Table 1:** Baseline characteristics of study population according to diagnosis of cancer

	Overall (*n* = 4140)	No cancer (*n* = 3698)	Cancer (*n* = 442)	*p* value
Sociodemographics				0.561
Age, years (mean, SD)	83.4 (9.4)	83.5 (9.5)	83.2 (9.0)	
Gender (no. %)				** < 0.001**
Female sex	3025 (73.1%)	2745 (74.2%)	280 (63.3%)	
Male sex	1115 (26.9%)	953 (25.8%)	162 (36.7%)	
Country (no. %)				** < 0.001**
Czech Republic	500 (12.1)	431 (11.7)	69 (15.6)	
Germany	493 (11.9)	442 (12)	51 (11.5)	
England	507 (12.2)	447 (12.1)	60 (13.6)	
Finland	478 (11.5)	446 (12.1)	32 (7.2)	
France	491 (11.9)	405 (11)	86 (19.5)	
Israel	580 (14)	540 (14.6)	40 (9.0)	
Italy	543 (13.1)	508 (13.7)	35 (7.9)	
Netherlands	548 (13.2)	479 (13)	69 (15.6)	
Comorbidities (no. %)				
Coronary heart disease	1064 (25.8)	954 (25.9)	110 (24.9)	0.654
Congestive heart failure	723 (17.5)	638 (17.3)	85 (19.2)	0.307
Diabetes mellitus	896 (21.7)	794 (21.5)	102 (23.1)	0.436
Stroke	909 (22.0)	813 (22.0)	96 (21.7)	0.890
Parkinson disease	290 (7.0)	260 (7.0)	30 (6.8)	0.847
Dementia	1492 (36.1)	1361 (36.9)	131 (29.6)	**0.003**
Depression^‡^	992 (23.9)	885 (23.9)	106 (23.6)	0.854
Chronic obstructive pulmonary disease	387 (9.3)	347 (9.4)	40 (9.0)	0.820
BMI (mean, SD)	24.56 (5.49)	24.58 (5.49)	24.43 (5.55)	0.600
< 18.0 (no.%)	375 (9.4)	326 (9.1)	49 (11.3)	0.148
Polypharmacy (no. %)	2972 (74.2)	2611 (73.1)	361 (83)	** < 0.001**
Cognitive impairment*				**0.010**
None	1949 (47.7)	1710 (46.8)	239 (54.4)	
Mild/moderate	879 (21.5)	794 (21.7)	85 (19.4)	
Severe	1262 (30.9)	1147 (31.4)	115 (26.2)	
ADL scale**				**0.012**
Independent	1274 (30.7)	1115 (30.2)	154 (34.8)	
Moderate dependent	1215 (29.4)	1111 (30.1)	104 (23.5)	
Severe dependent	1651 (39.9)	1467 (39.7)	184 (41.6)	
Self-rated health (no. %)				0.363
Excellent	238 (5.8)	216 (5.9)	22 (5.1)	
Good	932 (22.9)	828 (22.8)	104 (24)	
Fair	1211 (29.7)	1075 (29.6)	136 (31.3)	
Poor	542 (13.3)	477 (13.1)	65 (15)	
Not respond	1148 (28.2)	1041 (28.6)	107 (24.7)	
Symptoms (no. %)				
Delirium	482 (11.8)	428 (11.7)	54 (12.3)	0.718
Diarrhoea	450 (10.9)	393 (10.7)	57 (13)	0.143
Constipation	1091 (26.4)	967 (26.2)	124 (28.2)	0.372
Dizziness	1019 (24.7)	913 (24.7)	106 (24)	0.745
Falls	774 (18.9)	673 (18.4)	101 (23.1)	**0.016**
Pain	1492 (36.1)	1285 (34.8)	207 (46.8)	** < 0.001**
Dyspnoea	532 (12.9)	453 (12.3)	79 (17.9)	**0.001**
Insomnia	982 (23.8)	854 (23.2)	128 (29.2)	**0.005**
Fatigue	2036 (49.3)	1784 (48.4)	252 (57)	**0.001**
Advanced care directives (no. %)				
Do not intubate	284 (7.7)	239 (7.3)	45 (10.9)	**0.011**
Do not resuscitate	624 (17)	537 (16.5)	87 (21.1)	**0.019**
Do not hospitalize	270 (7.4)	218 (6.7)	52 (12.6)	** < 0.001**
No tube feeding	262 (7.1)	217 (6.7)	45 (10.9)	**0.002**

*p*-values for distribution of the variable between participants with and without cancer are in bold when signifcant at <0.05 level

*Mild/moderate cognitive impairment is defined by cognitive performance scale score (CPS) 2–4, severe impairment by CPS 5–6, none by CPS 0–1

**Assistance required is defined by ADL hierarchy scale score 3–4, dependent by ADL hierarchy scale score 5–6

^‡^Depression rating scale score ≥ 3

BMI, both as a continuous value and categorized, was similar among participants with and without cancer. Dementia was less prevalent in participants with cancer (29.6% vs. 36.9%, *p* = 0.003), and so was prevalence of cognitive impairment, according to the CPS scale. Polypharmacy was higher among participants with cancer (83% vs 73.1%, *p* = 0.003). Indicators of functional disabilities, such as ADL impairment, were significantly different among participants with and without cancer. Indicators of QoL, such as self-reported health and depression, were not significantly different among participants.

As shown in Table 1, falls, dyspnoea, insomnia and fatigue were all more prevalent in participants with cancer than in those without cancer: these differences were significant (23.1% vs 18.4; 17.9% vs 12.3%; 29.2% vs 23.2%; 57% vs 48.4%; *p* < 0.05 for all). Pain was also significantly more prevalent in participants with cancer than in participants without cancer (46.8% vs 34.8%; *p* < 0.001).

Advanced care directives such as do not intubate, do not resuscitate, do not hospitalize and no tube feeding were all more prevalent among participants with cancer (*p* < 0.05 for all): do not resuscitate directives, for instance, were present in 21.1% of participant with cancer vs 16.5% of participants without cancer.

**Fig. 1:**
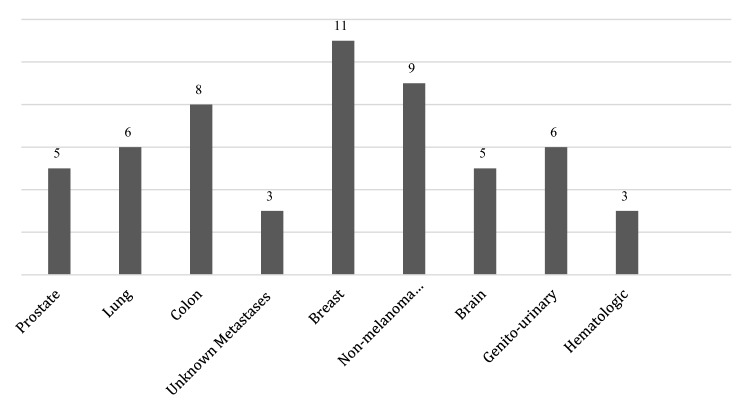
Primary tumour (available for 56 participants)

Primary tumour site was available for 56 participants. Breast cancer was the most prevalent, followed by non-melanoma skin cancers and colon cancer, as shown in Fig. 1.

Table 2 shows drug therapy and other treatments, according to cancer diagnosis. Regarding symptomatic drugs, NSAIDS, opioids, benzodiazepines and antidepressants were significantly more prescribed among participants with cancer (*p* < 0.05 for all), while paracetamol, antipsychotics and laxatives were not (*p* > 0.05 for all). Preventive drugs, such as acetylsalicylic acid, statins, vitamin D and bisphosphonates were all similarly prescribed among participants with and without cancer. Scheduled palliative care, wound care, oxygen therapy and ventilation were all more frequent among participants with cancer (*p* < 0.05 for all).

**Table 2:** Drugs and treatments according to diagnosis of cancer

	Overall (*n* = 4140)	No cancer (*n* = 3698)	Cancer (*n* = 442)	*p* value
NSAIDS	176 (4.4)	144 (3.9)	32 (7.2)	**0.001**
Paracetamol	920 (22.2)	806 (21.8)	114 (25.8)	0.056
Opioids	555 (13.4)	451 (12.2)	104 (23.5)	** < 0.001**
Laxatives	1675 (40.5)	1485 (40.2)	190 (43)	0.252
Antidepressants	1424 (34.4)	1251 (33.8)	173 (39.1)	**0.026**
Antipsychotics	1062 (25.7)	965 (26.1)	97 (21.9)	0.059
Benzodiazepines	1445 (34.9)	1267 (34.3)	178 (40.3)	**0.012**
Low-dose Acetylsalicylic acid	1514 (36.6)	1368 (37)	146 (33)	0.102
Statins	594 (14.3)	529 (14.3)	65 (14.7)	0.820
Vitamin D	163 (3.9)	142 (3.8)	21 (4.8)	0.352
Bisphosphonates	748 (18.1)	673 (18.2)	75 (17)	0.525
Palliative care	147 (3.6)	100 (2.7)	47 (10.7)	** < 0.001**
Oxygen therapy	66 (1.6)	45 (1.2)	21 (4.8)	** < 0.001**
Mechanical ventilation	26 (0.6)	18 (0.5)	8 (1.8)	**0.004**
Wound care	462 (11.2)	392 (10.7)	70 (15.9)	**0.001**

*p*-values for distribution of the variable between participants with and without cancer are in bold when signifcant at <0.05 level

Table 3 shows the results of the multivariable analyses. In the whole sample, male sex, polypharmacy, history of falls, pain and fatigue were associated to higher likelihood of presenting diagnosis of cancer, while dementia was associated to lower likelihood of presenting a cancer diagnosis. A sensitivity analysis was conducted to exclude those participants not receiving active treatment for cancer. The sensitivity analysis confirmed the direct association between polypharmacy and pain with cancer diagnosis, as well as the inverse association between female sex and dementia with cancer diagnosis. Moreover, the sensitivity analysis showed an inverse association between both mild/moderate and severe cognitive impairment with cancer and a direct association between severe dependency in ADLs and cancer.

**Table 3:** Factors associated with diagnosis of cancer

	Whole sample (*n* = 4140) OR (95% CI)	Excluding participants with cancer not receiving active treatment (*n* = 3896) OR (95% CI)
Age	1.00 (0.99–1.01)	0.99 (0.98–1.01)
Male sex	**1.67 (1.36–2.05)**	**1.61 (1.25–1.98)**
Polypharmacy	**1.59 (1.21–2.08)**	**1.86 (1.21–2.84)**
Cognitive impairment		
CPS 0–1	1	1
CPS 2–4	0.81 (0.61–1.09)	**0.54 (0.34–0.85)**
CPS 5–6	0.83 (0.62–1.11)	**0.63 (0.41–0.96)**
ADLs impairment		
ADL 0–1	1	1
ADL 2–4	0.72 (0.54–0.95)	1.19 (0.79–1.79)
ADL 5–6	1.19 (0.91–1.57)	**1.85 (1.24–2.78)**
Dementia	**0.74 (0.58–0.94)**	**0.51 (0.35–0.75)**
Falls	**1.30 (1.01–1.67)**	1.31 (0.91–1.89)
Pain	**1.43 (1.16–1.77)**	**1.41 (1.03–1.93)**
Dyspnoea	1.28 (0.97–1.69)	**1.50 (1.01–2.21)**
Fatigue	**1.25 (1.01–1.55)**	1.17 (0.85–1.60)

*p*-values for distribution of the variable between participants with and without cancer are in bold when signifcant at <0.05 level

## Discussion

The present study shows that cancer is a prevalent condition among European NHs residents and is associated to some specific characteristics. Yet, screening for asymptomatic cancer in older nursing home residents is not recommended in most countries [15]. The SHELTER population was mainly made up of women, but male sex showed a higher likelihood of having cancer, compared to female sex, as showed in the multivariable analysis. Prevalence of cancer patients in NHs varied considerably among different countries, ranging from 7.2% in Finland to 19.5% in France. The differences between the countries depend on many social and demographic factors, for instance countries such as the Netherlands offer the possibility to patients who are not completely independent to be assisted at home, even if they live on their own, which is not as feasible in Italy, possibly increasing the need of resorting to NHs [16]. It is important to underline that the prevalence of cancer in the SHELTER population is consistent with NHs literature. Anyway, prevalence of cancer is higher in the general elder population than in NHs [17]. Advanced care directives were all more prevalent among participants with cancer than those without it, which is interesting because in a recent study, more than a third of NH residents with advanced cancer experienced a potentially burdensome end-of-life transition, but not if they had advanced care directives [18].

In a German study including NHs residents, cancer was associated to a higher likelihood of having a BMI < 20, but not due to unintended weight loss or reduced caloric intake [19]. In our sample, no differences in BMI were found between participants with or without cancer, because the nutritional status of NHs residents is influenced by several different factors.

Cancer can affect cognition, either because of advanced disease or as a consequence of treatment (i.e. hormone therapy, chemotherapy, radiotherapy or brain surgery) [20]. In the present study, participant with cancer had a lower prevalence of dementia and, when excluding participants not receiving active cancer treatments, both mild/moderate and severe cognitive impairment were associated with a lower likelihood of cancer. This finding could be related to a selection bias, since cognitive impairment is one of the most common causes of admission to NHs [21]. Opposite results were found regarding functional impairment. When excluding participants not receiving active cancer treatments, severe dependency in the ADLs was associated with a higher likelihood of having cancer. Functional impairment of older adults with cancer can occur at any point in the diagnostic and therapeutic continuum: many patients who have been cured or have a long disease-free interval, for example, experience long-term treatment-related sequelae, that impair their functional status. The importance of functional measurement status predicts adverse effects (e.g. loss of independence, increased toxicity and reduced survival) associated to treatment [22, 23].

Falls and fatigue were significantly more prevalent in participants with cancer. Also, pain was more prevalent in participants with cancer, despite opioids and NSAIDS being more prescribed among participants with cancer. Symptom severity and symptom distress are aspects of the patients’ perspective that require assessment to warrant tailored management [24]. Pain still remains a common symptom among persons with cancer [25] and it has also been described that among NHs residents with cancer, pain was less frequently documented in those with severe cognitive impairment, raising the suspicion of lower pain treatment despite patients’ needs [26]. Therefore, a prerequisite to improve pain management is to understand patterns of pain and factors that may influence its onset and treatment.

Polypharmacy was prevalent both in participants with and without cancer, but it was significantly higher in those with cancer. This could be related to the need of taking drugs to treat symptoms associated with cancer. In fact there are evidences that older adults with cancer receive on average five ± four medications a day, increasing the risk of drug interactions and side effects. Because of this, there is an increased risk of falls, hospitalization, cognitive decline [27] and lower QoL [28]. In our study, symptomatic drugs such as NSAIDS, opioids, antidepressants and benzodiazepines are significantly more prescribed among participants with cancer. Also, preventive drugs such as low-dose acetylsalicylic acid and statins are widely prescribed among participants with and without cancer. These findings are similar to those of studies showing that patients with cancer in an acute hospital setting continued to receive preventive medications, and even have preventive medications started near end of life [29, 30]. Recent studies have shown that vitamin D supplementation in patients with cancer could have a positive impact [31]. But, in our sample, vitamin D prescription is low both in participants with and without cancer.

To manage NHs residents with cancer, it would be necessary to practice supportive care. Supportive care is an interdisciplinary medical specialty that focuses on preventing and relieving unnecessary suffering for patients facing serious and/or life-threatening illness, and it is not limited to the end-of-life care [32]. The primary goals of supportive care are symptom management and establishing plans of care in line with patients’ values and preferences. Within an integrated model of medical care in NHs, supportive care should be provided at the same time as curative and life-prolonging treatments, as well as in a palliative care setting [33].

### Limitations

The most important limitation of the present study is that InterRAI-LCTF is not specifically focused on collecting information about cancer. For this reason, aspects of cancer such as staging, time of diagnosis, and prognosis were not available. Also, it was possible to asses only 56 participants in terms of primary tumour site, which does not allow a thorough evaluation of the prevalence of different types of tumour. Another important limitation is that the present study is a secondary cross-sectional analysis of 10 year-old data. Still, the findings are consistent with literature and able to highlight several characteristics of NHs residents with cancer.

### Conclusions and implications

There is a high number of patients with cancer residing in NHs. Hence, NHs must adapt to provide cancer care. Although cancer patients who live in NHs have some similar characteristics to those who do not have cancer, they also have some peculiar characteristics, such as a lower prevalence of cognitive impairment and an increased symptomatic burden in term of fatigue and pain, which is not always properly managed. Further studies are needed to evaluate the impact of a supportive care approach on the management of NHs residents with cancer throughout all its phases, until the end-of-life care.

## Data Availability

Available.
